# Novel Entropy and Rotation Forest-Based Credal Decision Tree Classifier for Landslide Susceptibility Modeling

**DOI:** 10.3390/e21020106

**Published:** 2019-01-23

**Authors:** Qingfeng He, Zhihao Xu, Shaojun Li, Renwei Li, Shuai Zhang, Nianqin Wang, Binh Thai Pham, Wei Chen

**Affiliations:** 1 College of Geology & Environment, Xi’an University of Science and Technology, Xi’an 710054, Shaanxi, China; 2 State Key Laboratory of Geomechanics and Geotechnical Engineering, Institute of Rock and Soil Mechanics, Chinese Academy of Sciences, Wuhan 430071, Hubei, China; 3 Institute of Research and Development, Duy Tan University, Da Nang 550000, Vietnam

**Keywords:** rotation forest, credal decision tree, ensemble model, machine learning, landslide

## Abstract

Landslides are a major geological hazard worldwide. Landslide susceptibility assessments are useful to mitigate human casualties, loss of property, and damage to natural resources, ecosystems, and infrastructures. This study aims to evaluate landslide susceptibility using a novel hybrid intelligence approach with the rotation forest-based credal decision tree (RF-CDT) classifier. First, 152 landslide locations and 15 landslide conditioning factors were collected from the study area. Then, these conditioning factors were assigned values using an entropy method and subsequently optimized using correlation attribute evaluation (CAE). Finally, the performance of the proposed hybrid model was validated using the receiver operating characteristic (ROC) curve and compared with two well-known ensemble models, bagging (bag-CDT) and MultiBoostAB (MB-CDT). Results show that the proposed RF-CDT model had better performance than the single CDT model and hybrid bag-CDT and MB-CDT models. The findings in the present study overall confirm that a combination of the meta model with a decision tree classifier could enhance the prediction power of the single landslide model. The resulting susceptibility maps could be effective for enforcement of land management regulations to reduce landslide hazards in the study area and other similar areas in the world.

## 1. Introduction

Landslides, one of the most frequent geological hazards in China, cause thousands of millions of dollars in damage, dozens of casualties, and many geological environment problems every year [1,2,3,4,5]. In order to reduce the losses caused by landslides, predicting the areas where landslides are most likely to occur has become more important [3,6]. Landslide susceptibility research is an important approach to predicting the spatial distribution of landslides, which can be regarded as the spatial probability of landslide occurrence, according to a series of geoenvironmental conditions [7].

A landslide is a pattern of transforming the Earth’s surface under the influence of human activities [8,9,10]. Landslide is complex movement under the action of multiple factors, such as altitude, slope angle, rainfall, lithology, land use, and so on [11,12,13]. In recent years, more researchers have coupled geographic information systems (GISs) and assessment methods to study landslide susceptibility mapping, which has been confirmed to be effective [14,15,16]. 

As there are no universal methods in landslide susceptibility mapping, various approaches have been used to study landslide susceptibility, such as statistical models of entropy [17,18,19,20,21], evidential belief function [22,23], frequency ratio [24,25,26], weight of evidence [19,27], certainty factors [28,29,30], logistic regression models [31,32,33], and generalized additive models [34,35].

In addition to the above traditional statistical methods, various machine learning techniques have been introduced for landslide susceptibility mapping, such as artificial neural networks [24,36,37,38,39], support vector machines [34,40,41,42], naïve Bayes trees [43,44,45], alternating decision trees [46,47,48], rotation forests [32,49,50], kernel logistic regression [51,52], adaptive neuro-fuzzy inference systems [34,53,54], logistic model trees [49,52], and classification and regression trees [55,56,57]. However, the best method for landslide susceptibility mapping is still under discussion [58].

This paper takes Linyou County (China) as the study area and uses a novel, intelligent hybrid approach—a rotation-forest-based [59] credal decision tree classifier (RF-CDT) [60]—for landslide susceptibility mapping. In addition, two well-known ensemble models, bagging [61] and MultiBoostAB [62], were used as benchmark methods for comparison purposes. The results were validated by the area under the receiver operating characteristic (ROC) curve and statistical analysis. These landslide susceptibility maps were obtained in Linyou County and can be used for landslide mitigation and land use planning.

## 2. Study Area

**Figure 1:**
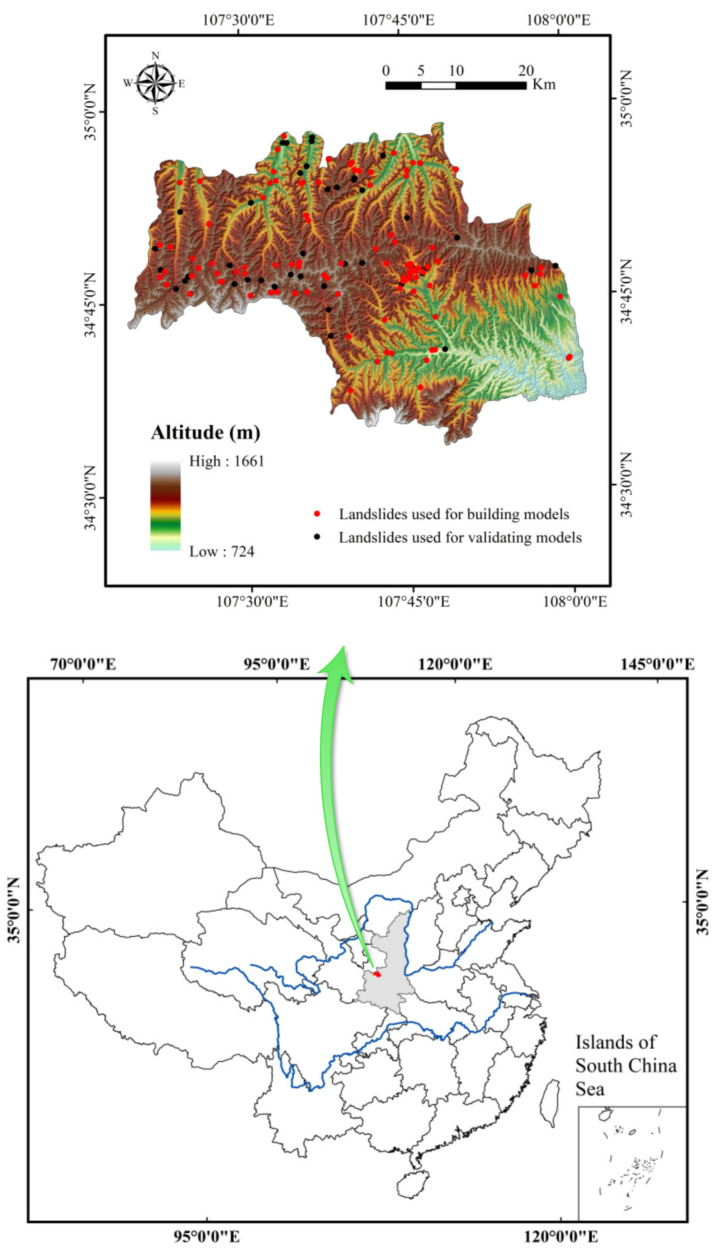
Study area.

Linyou County is located in the northeast of Baoji City in Shaanxi Province, China. It lies between longitudes of 107°19′–108°2′E and latitudes of 34°33′–34°58′N (Figure 1). Linyou County belongs to the temperate semihumid–humid monsoon climate zone. The climate is characterized by insufficient heat, droughty spring, cool summer, wet autumn, and cold winter. The average temperature is 9.1 °C. The annual average rainfall is 680 mm, mostly concentrated from July to September, accounting for more than 50% of the annual rainfall [63].

Topographically, the elevation increases from southeast to northwest, with average, highest, and lowest elevations of 1271, 1661, and 724 m, respectively. Slope angles of Linyou County range from 0 to 64.67°. Most of the slope angles are in the range of 10–20° (42.375%), followed by 20–30° (27.160%), 0–10° (22.910%), 30–40° (6.829%), 40–50° (0.700%), 50–60° (0.026%), and >60° (0.001%). Soil types are mainly Calcaric Cambisol (82.702%) and Eutric Cambisol (12.653%). 

## 3. Materials and Methods

### 3.1. Data Preparation

A landslide inventory map contains the previous locations of landslides [64]. In the current study, interpretations of multitemporal Google Earth data and historical records of landslides were used to prepare the primary landslide inventory map; furthermore, field surveys by handheld Global Positioning System (GPS) devices were carried out to verify landslide locations. Finally, a total of 152 landslides were mapped (Figure 1) and digitalized using ArcGIS software (Esri, Redlands, CA, USA) [65], including 113 slides and 39 falls [66], and were randomly divided into two parts (70/30) for the building and validation of models. 

The selection of conditioning factors is the foundation of landslide susceptibility assessment, and it has a direct impact on the evaluation results. However, there is no clear agreement with the precise cause of landslides due to their complex nature and development. Based on previous studies [67,68,69] and the geoenvironmental characteristics of the study area, 15 conditioning factors were selected: attitude, slope angle, slope aspect, plan curvature, profile curvature, sediment transport index (STI), stream power index (SPI), topographic wetness index (TWI), distance to rivers, distance to roads, normalized difference vegetation index (NDVI), soil, land use, lithology, and rainfall.

**Figure 2:**
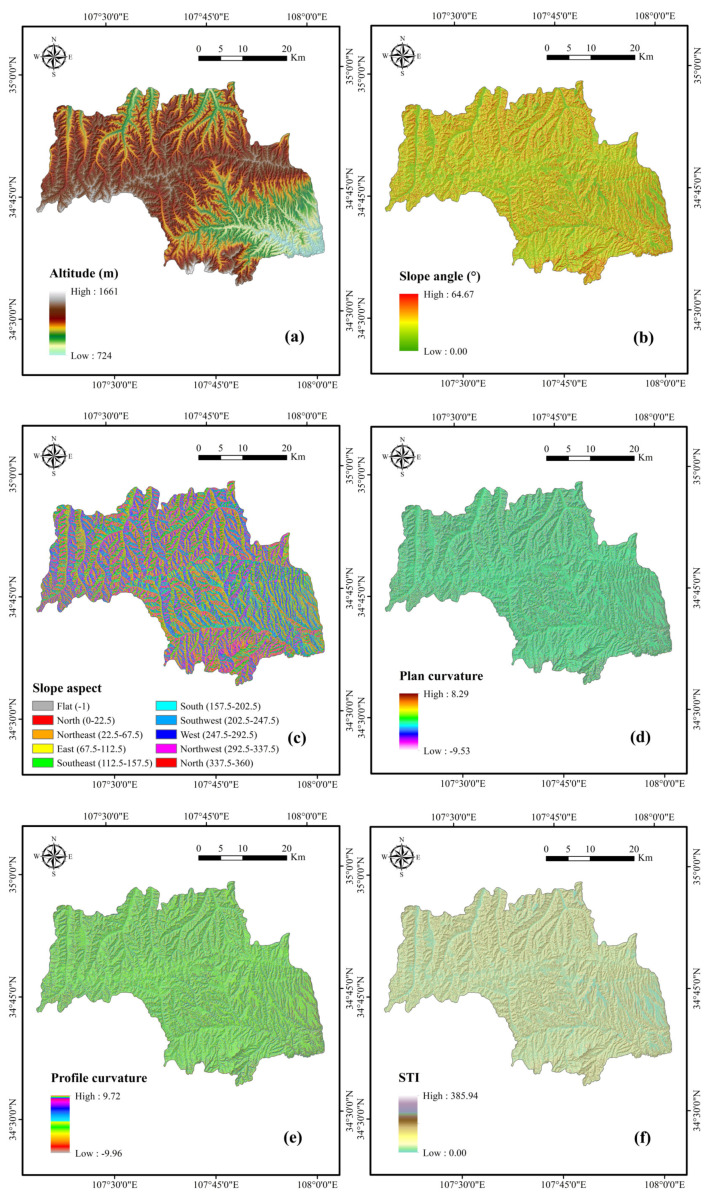

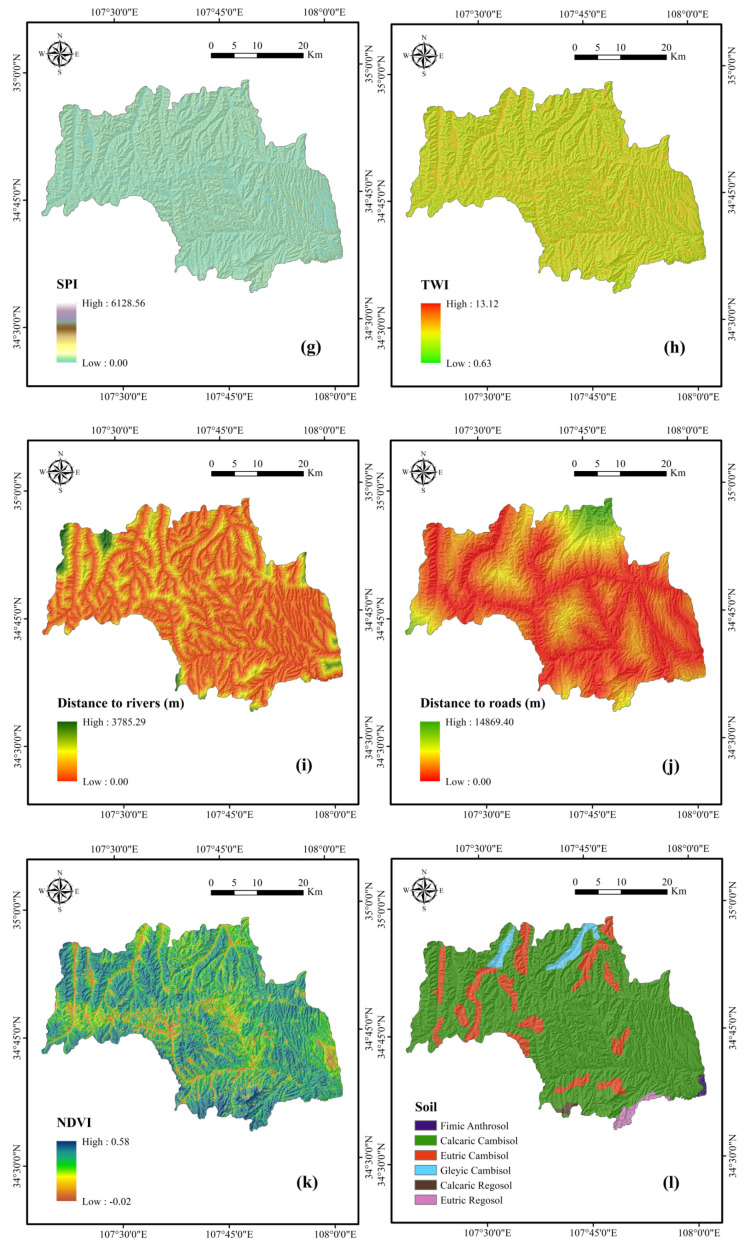

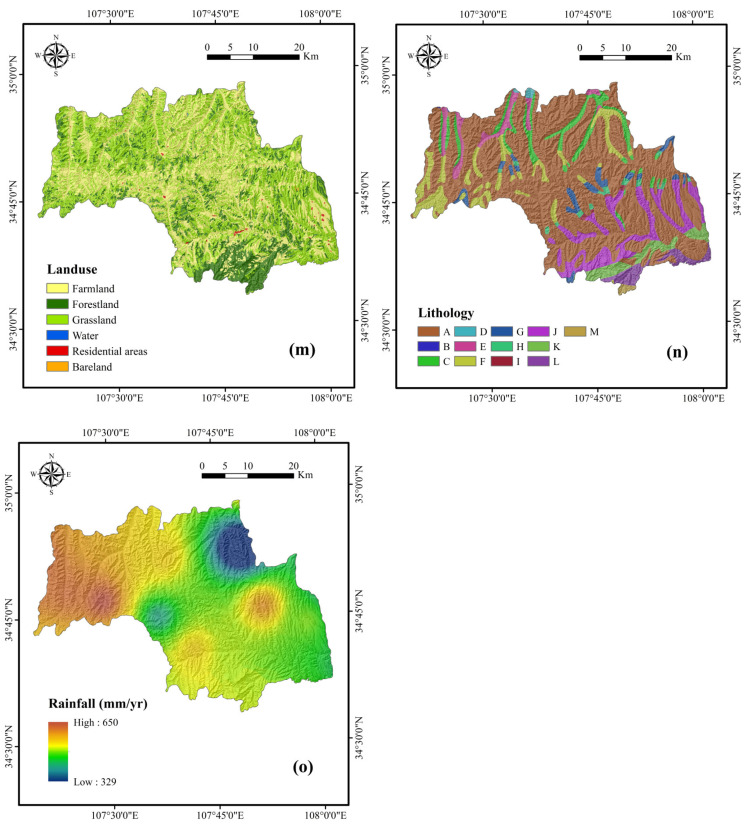
Thematic maps of the study area: (**a**) altitude; (**b**) slope angle; (**c**) slope aspect; (**d**) plan curvature; (**e**) profile curvature; (**f**) sediment transport index (STI); (**g**) stream power index (SPI); (**h**) topographic wetness index (TWI); (**i**) distance to rivers; (**j**) distance to roads; (**k**) normalized difference vegetation index (NDVI); (**l**) soil; (**m**) land use; (**n**) lithology; (**o**) rainfall.

Altitude, which greatly influences topographic attributes and controls differences in vegetation distribution, is one of the most commonly used factors in landslide susceptibility studies [70,71,72]. The altitude map (Figure 2a) was achieved from ASTER GDEM data with a resolution of 30 m collected from the National Aeronautics and Space Administration (NASA) [73]. In addition, DEM data were used to generate slope angle (Figure 2b), slope aspect (Figure 2c), plan curvature (Figure 2d), profile curvature (Figure 2e), STI (Figure 2f), SPI (Figure 2g), and TWI (Figure 2h) by GIS software [74,75]. 

Distances to rivers, which can influence the hydrologic processes of a slope, were obtained by buffering the river network from the topographic maps at the 1:50,000 scale (Figure 2i). Meanwhile, distances to roads were constructed by the same method from the road distribution maps (Figure 2j). This can be regarded as the impact of human activities on landslides, which causes a loss of toe support and changes the landform. NDVI is an index that shows the vegetation growth state and coverage. It can affect the stability of landslides through the reinforcement of plant roots and the permeability of surface soil (Figure 2k) [76,77,78].

The physical and mechanical properties of soil vary with soil type. They also influence the infiltration of surface water and the flow of ground water [79,80]. The soil types in the study area were classified into six classes (Figure 2l). Land use, an important conditioning factor in landslide susceptibility assessment, has been employed in many studies [81,82], and was classified into six types for this study (Figure 2m). Lithology is also a frequently used factor in landslide susceptibility analysis, because different rock strata have different physical and mechanical properties [67,83]. The lithology map was achieved from the geological maps at a scale of 250,000 and reclassified into 13 classes (Table 1, Figure 2n). Rainfall, widely considered as a controlling factor in landslide occurrence, can reduce the strength of rock and soil mass and increase slope weight [84,85,86]. The data were obtained from the Shaanxi Provincial Meteorological Bureau [87], and the maximum and minimum annual rainfall were 650 and 329 mm, respectively, in 2015 (Figure 2o).

### 3.2. Index of Entropy (IoE)

The entropy of a landslide refers to the extent to which various conditioning factors influence its development [20]. The equations used to calculate the information coefficient *W_j_* representing the weight values for the various conditioning factors [17,18] are as follows:

(1) $$P_{i}{}_{j}=\frac{Percentage\;of\;landslide}{Percentage\;of\;domain}$$

(2) $$(P_{ij})=\frac{P_{ij}}{\sum_{j=1}^{S_{j}}P_{ij}}$$

(3) $$H_{j\max}=\log_{2}S_{j},\;S_{j}\;\mathrm{is}\;\mathrm{the}\;\mathrm{number}\;\mathrm{of}\;\mathrm{classes}$$

(4) $$H_{j}=-\sum_{i=1}^{Sj}\left(P_{ij}\right)\log_{2}(P_{ij}),j=1,2,\ldots ,n$$

(5) $$I_{j}=\frac{H_{j\max}-H_{j}}{H_{j\max}}$$

(6) $$W_{j}=I_{j}\times P_{ij}$$

where *H_j_* and *H_jmax_* are the entropy values, *I_j_* is the information coefficient, and *W_j_* is the resulting weight value for the factors as a whole [21].

### 3.3. Credal Decision Tree

The credal decision tree (CDT) was proposed by Abellán and Moral in 2003 to address classification problems with credal sets [60]. During the construction process of a CDT, to avoid generating a too-complicated decision tree, a novel criterion was introduced: stop when the total uncertainty increases due to branching of the decision tree [88]. Based on Dempster’s and Shafer’s theory [89,90], an improved method was created to quantitatively measure the total uncertainty of credal sets. The function used in total uncertainty measurement can be briefly expressed as Equation (7):

(7) TU(ξ)=IG(ξ)+GG(ξ)

where *ξ* is a credal set on frame X, TU represents the value of total uncertainty, IG is a general function of nonspecificity on the corresponding credal set, and GG is a general function of randomness for a credal set. Abellán and Moral acquired sequences of conclusions and achievements related to total uncertainty measurement [91,92], and the calculation procedure of TU and properties of this measure are described systematically in relevant references. 

The imprecise Dirichlet model [93] was employed to compute the probability intervals of a variable. Suppose that *Z* is a variable whose values are represented by *z_j_*, and the corresponding probability distribution *p*(*z_j_*) satisfies Equation (8) [94]:

(8) $$p(z_{j})\in \left[\frac{n_{z_{j}}}{N+s},\frac{n_{z_{j}}+s}{N+s}\right]$$

where $n_{z_{j}}$ is the number of occurrences of the event where *Z* = *z_j_*, *N* is the sample size, and *S* is a hyperparameter whose value is usually 1 or 2, according to Walley [93].

### 3.4. Rotation Forest

Generally, it is considered that classifier ensembles can improve the performance of a single classifier [59]. As a novel technique to construct classifier ensembles, the rotation forest (RF) model has been widely used in landslide susceptibility mapping with the aim of acquiring better prediction accuracy [95,96,97]. Suppose that *X* is the original training data, and *X* can be written as an *N* × *n* matrix (*N* is the number of training samples, and *n* is the number of features). The corresponding class label set and feature set can be denoted as *Y* and *F*, respectively. Assume that *L* is the total number of decision tree classifiers in the RF algorithm, and the *i*th decision tree is *Di* (*i* = 1, 2, …, *L*). In the RF algorithm, *F* is first randomly split into *k* subsets. We can then obtain *F_ij_* (the *j*th feature subset for the *i*th decision tree) and *X_ij_* (the training data for features in *F_ij_*). Based on the bootstrap approach, a nonempty subset $X_{ij}^{\prime }$ is generated, whose size is 75% of the original training data. In the next step, an *M* × 1 (*M* = *n*/*k*) coefficient vector is obtained by using linear transformation on $X_{ij}^{\prime }$, and the coefficient vector can be expressed as $\left\{a_{ij}^{1},\ldots ,a_{ij}^{M1}\right\}$. Subsequently, a sparse rotation matrix *R_i_* can be created, shown as Equation (9):

(9) $$R_{i}=\left[\begin{array}{cccc} a_{i1}^{\left(1\right)},\ldots ,a_{i1}^{\left(M1\right)} & \left\{0\right\} & \ldots & \left\{0\right\}\\ \left\{0\right\} & a_{i2}^{\left(2\right)},\ldots ,a_{i2}^{\left(M2\right)} & \ldots & \left\{0\right\}\\ \vdots & \vdots & \vdots & \vdots \\ \left\{0\right\} & \left\{0\right\} & \ldots & a_{ik}^{\left(k\right)},\ldots ,a_{ik}^{\left(Mk\right)} \end{array}\right].$$

In this way, the new training dataset for *D_i_* can be calculated as Equation (4), and all the single decision tree classifiers will be trained in a parallel manner [98].

(10) $$\mathrm{Transformed}\;\mathrm{training}\;\mathrm{set}=XR_{i}^{a}$$

where $R_{i}^{a}$ is the new sparse rotation matrix formed by rearranging the columns of *R_i_* according to the original feature set.

### 3.5. Bagging

Bagging is an abbreviation for “bootstrap aggregating”, which is a technique to raise the accuracy of machine learning algorithms [61]. The main idea of bagging is that it generates an ensemble classifier composed of multiple base classifiers that are constructed with various bootstrapped training sets [99]. Bagging not only contributes to decreasing the classification variance but also can improve the generalization capability of the ensemble classifier [61]. It has been proved that the combining rule of base classifiers may have a notable effect on bagging performance [100]. Currently, the majority vote combining rule has been adopted extensively in bagging. The ultimate classification result can be obtained by the formula demonstrated in Equation (11):

(11) $$C^{*}(x)=\underset{y\in Y}{\arg\max}\sum_{i=1}^{t}1(C_{i}(x)=y)$$

where $1(C_{i}(x)=y)$ is the indicator function.

### 3.6. MultiBoostAB

MultiBoostAB is the Waikato Environment for Knowledge Analysis (WEKA) version of MultiBoosting [62]. In essence, MultiBoosting is a combination of AdaBoost and wagging, a variant of bagging [101]. AdaBoost and bagging are two widely used techniques in the field of ensemble learning [96,99,102]. It was demonstrated that AdaBoost could remarkably decrease the bias and variance of classifiers, while bagging only had an attenuation effect on variance [103]. However, it has been proved that bagging has better performance in error reduction [61]. Compared with bagging, wagging determines random instance weights with the continuous Poisson distribution. Suppose that *i* is the number of subcommittees, *I_i_* is a variable to limit the iterations of the *i*th subcommittee, and *T* represents the number of iterations. Values of *I_i_* can be calculated by Equation (12):

(12) $$\{\begin{array}{c} n=\left\lfloor T\right\rfloor \\ I_{i}=\left\lceil i\times T/n\right\rceil \;(i=1,2,\cdots ,n-1)\\ I_{i}=T\;(i=n,n+1,\cdots ,\infty ) \end{array}$$

In the process of iteration, the weighted errors on training sets can be figured out by Equation (13). *β_t_* depends on the corresponding value of error, and the final classification function is shown as Equation (14) [101]:

(13) $$\varepsilon_{t}=\frac{\sum_{x_{j}\in S^{\prime }:C_{t}(x_{j})\neq y_{j}}weight(x_{j})}{m}$$

(14) $$C^{*}(x)=\underset{y\in Y}{\arg\max}\sum_{t:C_{t}(x)=y}\log\frac{1}{\beta_{t}}$$

where *ε_t_* refers to the weighted error, *m* is the number of examples in the training sequence, and *C_t_*(*x_j_*) is the classification result of the *t*th base classifier.

## 4. Results and Analysis

### 4.1. Selection of Landslide Conditioning Factors

In the present study, the index of entropy model was used to reduce the unevenness among the factors and thereby provide a realistic status of their impact on landslide susceptibility (Table 2) [104]. The results of each class of the conditioning factors were then extracted as inputs to calculate the importance of conditioning factors and modeling landslide susceptibility. The result of the importance of conditioning factors by correlation attribute evaluation (CAE) [105] is shown in Table 3. It shows that all the conditioning factors contribute to the landslide susceptibility model. NDVI, with an average merit (AM) of 0.273, has the highest AM of all the conditioning factors, followed by distance to roads (AM = 0.242), land use (AM = 0.191), distance to rivers (AM = 0.127), rainfall (AM = 0.092), STI (AM = 0.091), SPI (AM = 0.090), profile curvature (AM = 0.072), plan curvature (AM = 0.060), lithology (AM = 0.055), TWI (AM = 0.048), soil (AM = 0.044), slope aspect (AM = 0.025), slope angle (AM = 0.015), and altitude (AM = 0.014). All 15 conditioning factors were applied to create the landslide susceptibility maps in the study area in virtue of their positive contributions to the models.

### 4.2. Generation of Landslide Susceptibility Maps

**Figure 3:**
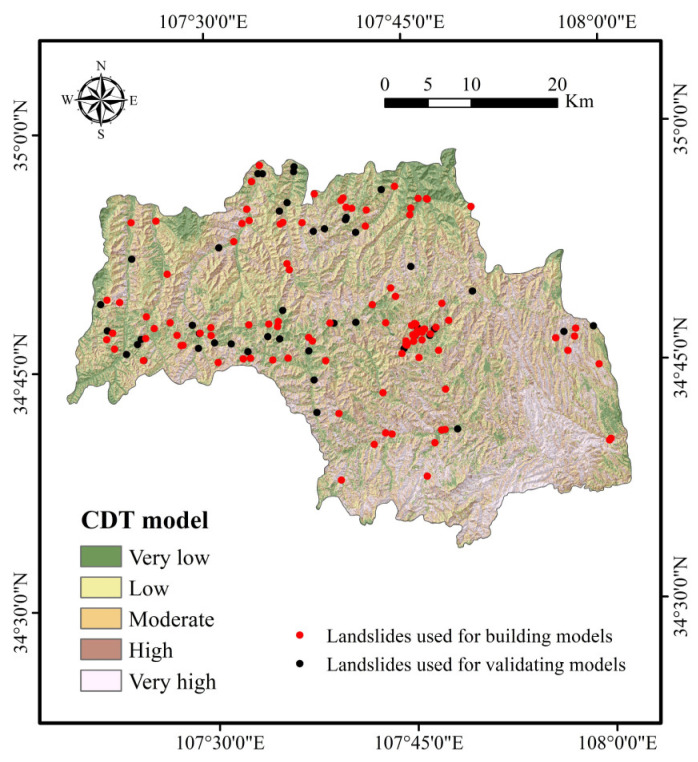
Landslide susceptibility map using the credal decision tree (CDT) model.

**Figure 4:**
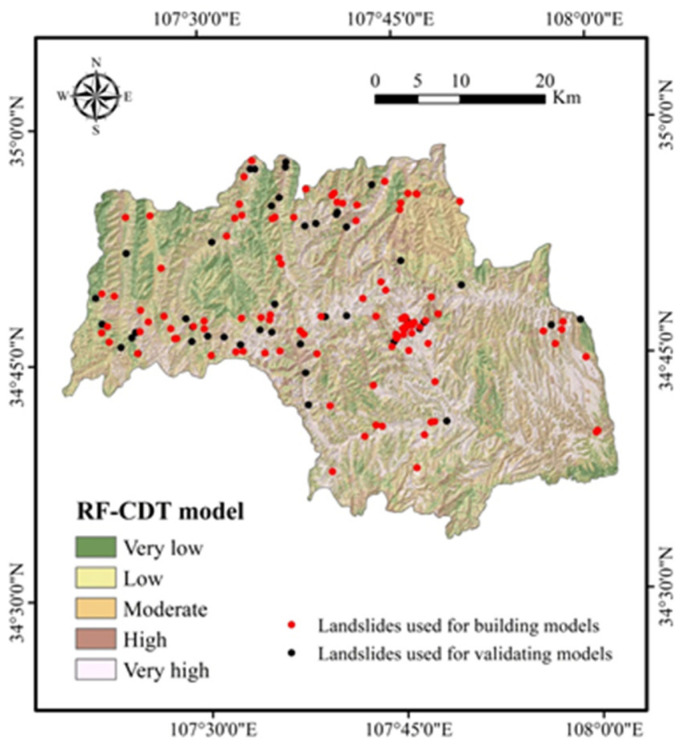
Landslide susceptibility map using the rotation forest (RF)-CDT model.

After the training and validation processes of landslide models, landslide susceptibility maps were obtained in the following two steps. First, the probability of landslide occurrence (PLO) for each pixel was generated using the probability distribution functions of the CDT and RF-CDT models. In the second step, PLOs were reclassified by mathematical methods, such as standard deviation, equal interval, natural break, geometric interval, and quantile. In this study, the quantile method was exploited to divide the PLOs into five categories: very low, low, moderate, high, and very high. The quantile method is a standard classification method in ArcGIS software that provides a more comprehensive analysis for both linear and nonlinear models in practical problems and makes a useful supplement for general regression models [106,107]. Therefore, the landslide susceptibility mappings (LSMs) in this research were classified by the quantile method. Figure 3 and Figure 4 present the results of LSMs for the CDT and RF-CDT models, respectively. 

**Figure 5:**
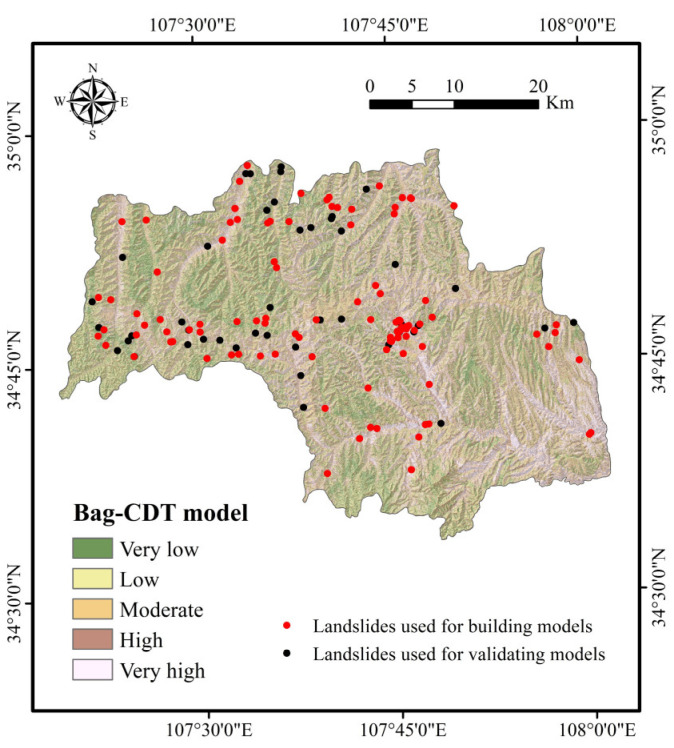
Landslide susceptibility map using the bag-CDT model.

**Figure 6:**
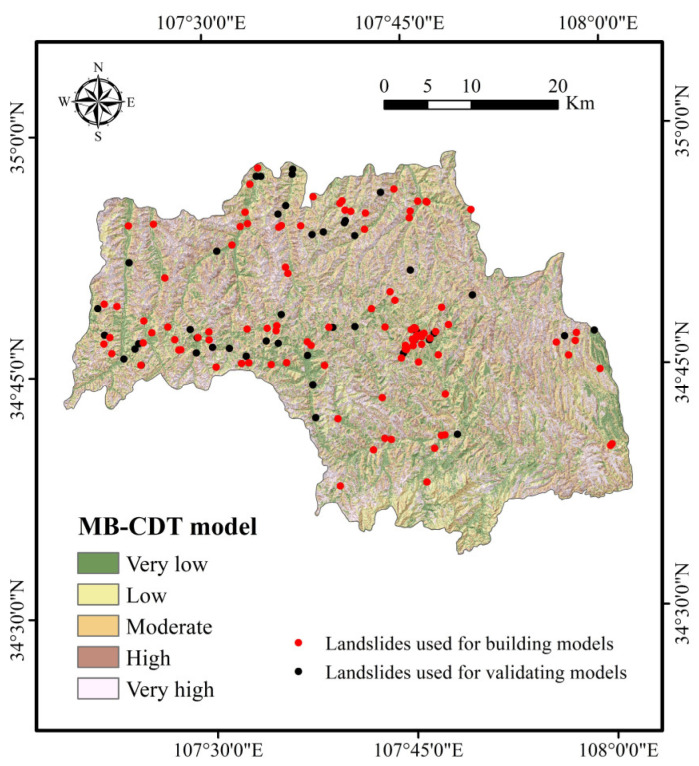
Landslide susceptibility map using the MultiBoostAB (MB)-CDT model.

**Figure 7:**
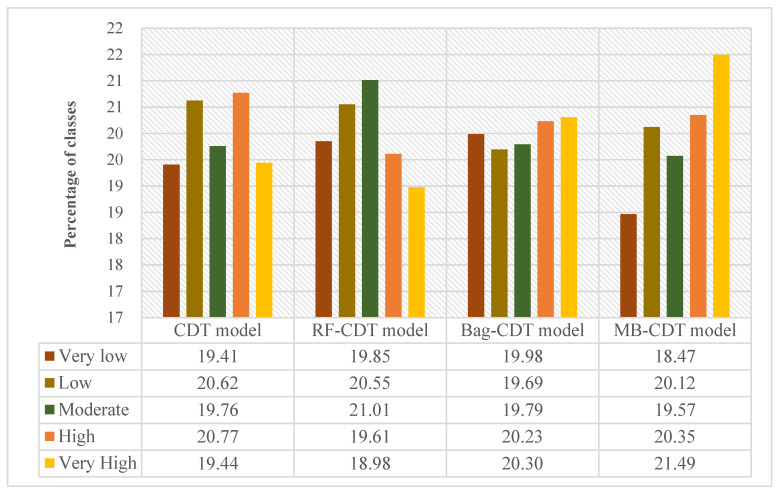
Area percentages of landslide susceptibility classes.

To further demonstrate the feasibility of the RF-CDT model in the landslide susceptibility study, two ensemble models, consisting of the CDT model as well as bagging and MultiBoostAB, were introduced to the benchmark models. The establishment, training, validation, and assessment processes of the benchmark models were the same as with the RF-CDT model, and landslide susceptibility maps generated by the benchmark models are shown in Figure 5 and Figure 6. Area percentages of landslide susceptibility classes of all models are shown in Figure 7.

### 4.3. Model Validation and Comparison

**Figure 8:**
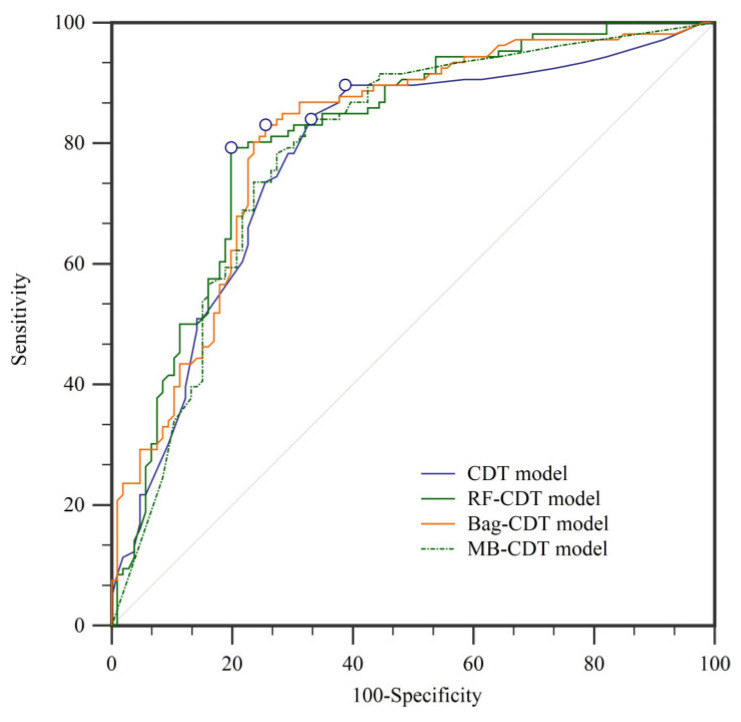
Receiver operating characteristic (ROC) curves using training dataset.

**Figure 9:**
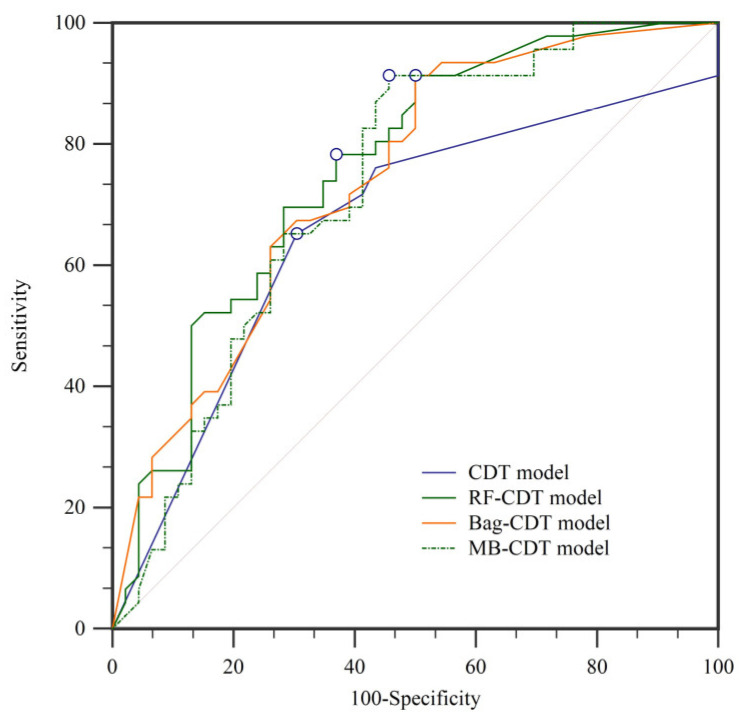
ROC curves using validation dataset.

In landslide susceptibility modeling, it is essential to validate and compare the quality of results. Validation of the results is regarded as one of the most important aspects of landslide susceptibility research, and the assessment results will not show scientific significance without validation [34,108]. In this paper, the prediction ability of the four models was evaluated using the receiver operating characteristic (ROC) curve [109,110]. The ROC curves and the parameters of the ROC curves using the training dataset are shown in Figure 7 and Table 4, respectively. Similarly, the ROC curves and the parameters of the ROC curves using the validation dataset are shown in Figure 8 and Figure 9 and Table 5, respectively. In the training dataset, the RF-CDT model has the highest area under the ROC curve (AUC) value (0.813), followed by the bag-CDT model (0.809), the MB-CDT model (0.788), and the CDT model (0.779). The model with the highest AUC value for the validation dataset was RF-CDT (0.759), followed by bag-CDT (0.740), MB-CDT (0.729), and CDT (0.663). It can be concluded that the RF-CDT model had the best performance in both training and validation processes. All the evaluation results were obtained under a confidence interval (CI) at 95%.

## 5. Discussion

Landslides have caused much financial loss and have threatened the safety of humans all over the world [111]. Various approaches have been used to study landslide susceptibility, and the research methods have evolved from simple statistical models to machine learning models. In order to achieve precise evaluation results, the use of new models in landslide susceptibility research has become more important. In this study, we chose the credal decision tree (CDT) as the basic model and combined it with rotation forest (RF), bagging (bag), and MultiBoostAB (MB) models to build ensemble models.

As there are no standards for selecting landslide conditioning factors [112], how to determine the conditioning factors has become a very important issue. In order to deal with it reasonably, the selection of conditioning factors in this paper was based on the geoenvironmental characteristics of the study area, the mechanism of landslide occurrence, and similar landslide susceptibility studies. 

According to the importance analysis by the CAE model, it can be concluded that the NDVI, a commonly used conditioning factor that indicates the state of plant growth in the study area, is the most important landslide conditioning factor. According to its definition, the interval of NDVI value is [−1, 1] and the higher the value, the better the vegetation growth. The study area lies in hilly and valley regions of the Weibei dry plateau, one of the key areas of soil and water loss of Shaanxi Province, and rainfall is mainly concentrated from July to September. Therefore, under the joint action of uneven distribution of rainfall and serious soil erosion, the vegetation growth of the study area is relatively low, and the NDVI interval is [−0.09, 0.39]. In addition, many studies have indicated that plants play a positive role in landslide occurrence because their root systems can increase soil strength and reduce water infiltration [113,114,115]. 

In the case of land use, the average merit is 0.191. It is well known that land use has a close relationship with human activities and may affect soil and water loss, precipitation infiltration, and surface structure [116]. It can be seen in Figure 2m that farmland is the main type of land use. As the study area is located in the Weibei dry plateau, the infiltration of agricultural water will increase slope mass and reduce soil strength, which makes landslides occur more easily. It can be seen in Figure 4, Figure 5, Figure 6 and Figure 7 that most landslides occur in low-altitude areas with nearby linear conditioning factors, such as distance to roads and rivers. Correspondingly, we can find that landslides decrease as we move away from roads and rivers. These results can also be found in similar studies [117,118].

According to the parameters of ROC curves of the training and validation datasets, the RF-CDT model reflected the spatial distribution of landslides perfectly, while the CDT model had the lowest accuracy rate. The rotation forest model is a powerful new machine learning method that has been widely used in many fields and performed admirably in previous landslide susceptibility studies [32,49,119]. The bag-CDT model performed worse than the RF-CDT model, and its AUC values of training and validation datasets were 0.809 and 0.740, respectively. The MB-CDT model ranked third, with training and validation dataset AUC values of 0.788 and 0.729, respectively. 

In a nutshell, the ensemble models in this paper expressed more promising results compared to single evaluation models in current studies [96,120,121]. Based on the CDT model combined with the RF, bag, and MB models, landslide susceptibility in Linyou County was studied. As mentioned above, the RF-CDT model performed best in this research compared to other models. This raised a question as to why AUC values increased rapidly with the CDT model combined with the RF model. Perhaps the answer to this question can be explained as “slightly underperformed,” which means that there should be a threshold for positive synergy among models [122,123]. In this paper, the RF model had the best cooperation with the CDT model. However, limits in different models have different interconnection rules that may be difficult to determine, especially when facing a series of factors with various ranges.

## 6. Conclusions

The present study allowed us to reach the following conclusions:

(1) The importance of conditioning factors was quantitatively defined by CAE. All 15 conditioning factors were applied to create the landslide susceptibility maps, and NDVI had the highest importance of all the conditioning factors.

(2) The proposed hybrid RF-CDT model, with AUC values of 0.813 and 0.759, achieved good results in the training and validation phases compared to the single CDT model. 

(3) The performance of the proposed hybrid RF-CDT model was also compared with the hybrid bag-CDT and MB-CDT models, and the results of AUC, SE, and CI at 95% also indicate that the RF-CDT model is a promising method.

As a final remark, it is worth noting that the present study indicates that machine learning ensemble frameworks are promising techniques, and the obtained susceptibility maps may be employed to manage land use planning and landslide risk mitigation.

## Figures and Tables

**Table 1:** Lithology of the study area.

Name	Lithology	Geological Age
Group A	Loess	Quaternary
Group B	Gravel, fine sandstone, argillaceous silt	Quaternary
Group C	Brown-red calcareous clay rock interbedded with sandy clay rock, sandstone, and glutenite	Neogene
Group D	Sandstone interbedded with mudstone; mudstone and siltstone interbedded with sandstone	Cretaceous
Group E	Powder-fine sandstone, mudstone interbedded with tuff and marlstone	Cretaceous
Group F	Sandstone interbedded with conglomerate	Cretaceous
Group G	Conglomerate interbedded with glutenite and sandstone	Cretaceous
Group H	Feldspathic sandstone, mudstone, siltstone, coarse sandstone, fine conglomerate	Jurassic
Group I	Interbedded sandstone and mudstone, coarse sandstone, sandstone, coal seam	Jurassic
Group J	Interbedded sandstone and mudstone, marlstone, conglomerate, sandstone, siltstone, shale, oil shale	Triassic
Group K	Sandstone interbedded with mudstone, siltstone, and coal seam	Permian
Group L	Conglomerate, siliceous dolomite, shale, shale interbedded with sandstone	Ordovician
Group M	Upper: argillaceous dolomite Middle: fine-grained dolomite Bottom: spatulate dolomite, oolitic dolomite	Cambrian

**Table 2:** Correlation between landslides and conditioning factors using the index of entropy (IoE) method.

Conditioning Factor	Classes	Percentage of Domain	Percentage of Landslides	(P_ij_)	I_j_	W_j_
Altitude (m)	724–800	0.103	0.000	0.000	0.203	0.168
	800–900	0.779	1.887	0.292		
	900–1000	2.705	0.000	0.000		
	1000–1100	7.581	6.604	0.105		
	1100–1200	14.306	21.698	0.183		
	1200–1300	24.928	27.358	0.132		
	1300–1400	30.262	21.698	0.086		
	1400–1500	17.504	19.811	0.136		
	1500–1600	1.733	0.943	0.066		
	1600–1661	0.099	0.000	0.000		
Slope angle (°)	0–10	22.910	23.585	0.244	0.229	0.162
	10–20	42.375	41.509	0.232		
	20–30	27.160	26.415	0.230		
	30–40	6.829	8.491	0.294		
	40–50	0.700	0.000	0.000		
	50–64.67	0.027	0.000	0.000		
Slope aspect	Flat	0.028	0.000	0.000	0.095	0.085
	North	11.352	6.604	0.072		
	Northeast	13.563	10.377	0.094		
	East	14.844	16.038	0.133		
	Southeast	11.877	22.642	0.235		
	South	10.414	14.151	0.168		
	Southwest	12.378	15.094	0.151		
	West	13.614	7.547	0.068		
	Northwest	11.928	7.547	0.078		
Plan curvature	Concave	45.118	34.906	0.240	0.020	0.021
	Plan	8.877	11.321	0.396		
	Convex	46.005	53.774	0.363		
Profile curvature	Concave	45.281	48.113	0.361	0.002	0.002
	Plan	7.095	6.604	0.316		
	Convex	47.624	45.283	0.323		
STI	<10	76.576	82.075	0.324	0.345	0.228
	10–20	17.018	12.264	0.218		
	20–30	3.726	5.660	0.459		
	30–40	1.317	0.000	0.000		
	>40	1.363	0.000	0.000		
SPI	<10	56.676	59.434	0.223	0.054	0.051
	10–20	19.037	23.585	0.263		
	20–30	7.932	2.830	0.076		
	30–40	4.124	5.660	0.291		
	>40	12.230	8.491	0.147		
TWI	<2	56.140	62.264	0.332	0.160	0.107
	2–3	35.052	31.132	0.266		
	3–4	6.804	5.660	0.249		
	4–5	1.845	0.943	0.153		
	>5	0.159	0.000	0.000		
Distance to rivers (m)	<200	26.385	28.302	0.219	0.018	0.017
	200–400	22.387	28.302	0.258		
	400–600	17.492	19.811	0.231		
	600–800	12.379	9.434	0.156		
	>800	21.357	14.151	0.135		
Distance to roads (m)	<500	16.524	27.358	0.299	0.036	0.040
	500–1000	14.614	20.755	0.257		
	1000–1500	12.738	9.434	0.134		
	1500–2000	10.994	11.321	0.186		
	>2000	45.130	31.132	0.125		
NDVI	−0.02–0.23	7.755	16.981	0.288	0.216	0.328
	0.23–0.32	10.093	28.302	0.369		
	0.32–0.38	18.757	41.509	0.291		
	0.38–0.44	34.724	11.321	0.043		
	0.44–0.58	28.672	1.887	0.009		
Soil	Fimic Anthrosol	0.328	0.000	0.000	0.436	0.325
	Calcaric Cambisol	82.702	79.245	0.214		
	Eutric Cambisol	12.653	14.151	0.250		
	Gleyic Cambisol	2.750	6.604	0.536		
	Calcaric Regosol	0.377	0.000	0.000		
	Eutric Regosol	1.190	0.000	0.000		
Land use	Farmland	34.928	65.094	0.282	0.477	0.525
	Forestland	16.617	0.943	0.009		
	Grassland	48.185	33.019	0.104		
	Water	0.008	0.000	0.000		
	Residential areas	0.236	0.943	0.605		
	Bareland	0.025	0.000	0.000		
Lithology	A	65.720	52.830	0.043	0.239	0.343
	B	0.021	0.000	0.000		
	C	5.811	5.660	0.052		
	D	0.251	0.943	0.201		
	E	3.165	9.434	0.160		
	F	7.254	7.547	0.056		
	G	2.576	12.264	0.255		
	H	0.965	2.830	0.157		
	I	0.245	0.000	0.000		
	J	8.257	6.604	0.043		
	K	3.074	1.887	0.033		
	L	2.336	0.000	0.000		
	M	0.326	0.000	0.000		
Rainfall (mm/yr)	<400	2.041	0.000	0.000	0.210	0.161
	400–500	7.117	6.604	0.303		
	500–600	74.158	74.528	0.328		
	>600	16.684	18.868	0.369		

**Table 3:** Importance of conditioning factors based on correlation attribute evaluation (CAE).

Landslide Conditioning Factor	Average Merit (AM)	Standard Deviation (SD)
NDVI	0.273	±0.019
Distance to roads	0.242	±0.014
Land use	0.191	±0.020
Distance to rivers	0.127	±0.019
Rainfall	0.092	±0.017
STI	0.091	±0.026
SPI	0.090	±0.032
Profile curvature	0.072	±0.017
Plan curvature	0.060	±0.023
Lithology	0.055	±0.015
TWI	0.048	±0.021
Soil	0.044	±0.016
Slope aspect	0.025	±0.017
Slope angle	0.015	±0.015
Altitude	0.014	±0.010

**Table 4:** Parameters of ROC curves using training dataset. AUC: area under the receiver operating characteristic curve; SE: standard error; CI: confidence interval.

Model	AUC	SE	95% CI
CDT	0.779	0.0328	0.717 to 0.833
RF-CDT	0.813	0.0300	0.754 to 0.863
Bag-CDT	0.809	0.0302	0.750 to 0.860
MB-CDT	0.788	0.0320	0.727 to 0.841

**Table 5:** Parameters of ROC curves using validation dataset.

Model	AUC	SE	95% CI
CDT	0.663	0.0547	0.557 to 0.758
RF-CDT	0.759	0.0504	0.658 to 0.842
Bag-CDT	0.740	0.0515	0.638 to 0.826
MB-CDT	0.729	0.0537	0.626 to 0.816
